# Clinical characteristics and prognosis of patients with left ventricular thrombus in East China

**DOI:** 10.3389/fcvm.2022.944687

**Published:** 2022-09-08

**Authors:** Cheng Li, Wenjie Lau, Ningjing Qian, Liuguang Song, Chunna Jin, Dao Zhou, Yi Yu, Xiaohong Pan, Quan Zhou

**Affiliations:** ^1^Nursing Department, The Second Affiliated Hospital of Zhejiang University School of Medicine, Hangzhou, China; ^2^Department of Cardiology, Second Affiliated Hospital, School of Medicine, Zhejiang University, Hangzhou, China; ^3^Department of Pharmacy, Second Affiliated Hospital, School of Medicine, Zhejiang University, Hangzhou, China

**Keywords:** left ventricular thrombus, clinical characteristics, treatment, prognosis, MACE, bleeding

## Abstract

**Background:**

Left ventricular thrombus (LVT) is a serious complication in patients with left ventricular dysfunction. However, there is still a paucity of data on treatments and prognosis of patients with LVT. This study aims to evaluate the clinical characteristics of patients with LVT and to determine the impact of LVT on the incidence of major adverse cardiovascular events (MACEs) and all-cause mortality.

**Methods:**

From January 2010 to January 2020, 237 patients diagnosed with LVT at The Second Affiliated Hospital Zhejiang University School of Medicine in East China were retrospectively included. Clinical characteristics, treatments, MACEs, and bleeding events [thrombolysis in myocardial infarction (TIMI) I and II] were collected. MACE is determined as the composite of all-cause mortality, ischemic stroke, acute myocardial infarction (MI), and acute peripheral artery emboli.

**Results:**

The all-cause mortality rate was 28.3% (89.6% due to cardiovascular death), ischemic stroke 8.4%, MI 3%, peripheral artery emboli 1.7%, and bleeding events (TIMI I and II) 7.6% were found during a median follow-up of 736 days. Total LVT regression occurred in 152 patients (64.1%). Atrial fibrillation [hazard ratio (HR), 3.049; 95% confidence interval (95% CI) 1.264–7.355; *p* = 0.013], moderate and severe renal function injuries (HR, 2.097; 95% CI, 1.027–4.281; *p* = 0.042), and left ventricular ejection fraction (LVEF) ≤ 50% (HR, 2.243; 95% CI 1.090–4.615; *p* = 0.028) were independent risk factors for MACE, whereas the use of β-blocker (HR, 0.397; 95% CI 0.210–0.753; *p* = 0.005) was its protective factor. Age (HR, 1.021; 95% CI 1.002–1.040; *p* = 0.031), previous caronary artery bypass grafting (CABG; HR, 4.634; 95% CI 2.042–10.517; *p* < 0.001), LVEF ≤ 50% (HR, 3.714; 95% CI 1.664–8.290; *p* = 0.001), and large thrombus area (HR, 1.071; 95% CI 1.019–1.126; *p* = 0.007) were independent risk factors for increasing all-cause mortality, whereas the use of β-blocker (HR, 0.410; 95% CI 0.237–0.708; *p* = 0.001) was protective factor.

**Conclusion:**

This study showed that atrial fibrillation, moderate and severe renal dysfunction, and LVEF ≤ 50% were independent risk factors for MACE; age, previous CABG, LVEF ≤ 50%, and large thrombus area were independent risk factors for all-cause mortality. It was found that the use of β-blockers could improve the prognosis of patient with LVT for the first time. It is recommended that clinicians could be more active in applying patient with LVT with anticoagulants.

## Introduction

Left ventricular thrombus (LVT) is a serious complication in patients with left ventricular dysfunction and is associated with poor outcomes. Despite adequate interventional and medical therapy, LVT remains to be an important source of cerebral and peripheral arterial embolism with a subsequent increased mortality (1, 2). Heart diseases, such as acute myocardial infarction (MI), cardiomyopathy, valvular heart disease, myocarditis, and myocardial insufficiency, are common causes of LVT. According to a single-center retrospective study from May 2003 to November 2011, the population incidence rate of LVT was 0.72‰. Coronary heart disease is the main cause (80.6%). Other causes of LVT include dilated cardiomyopathy (DCM) 8.1%, hypertrophic cardiomyopathy 3.2%, stress cardiomyopathy 4.8%, aortic valve stenosis 1.6%, and Brugada syndrome 1.6% (3). To date, clinical features, treatments, and the prognosis of acute MI-related LVT have been well studied, but the follow-up time was relatively short. Besides that, the prognosis of other disease-related LVT, such as cardiomyopathy, valvular disease, and myocarditis, is still rarely reported, nationally and internationally. In this study, we retrospectively analyzed the clinical characteristics, treatments, and the prognosis of LVT from a comprehensive spectrum of diseases during a 1- to 10-year follow-up from The Second Affiliated Hospital Zhejiang University School of Medicine (SAHZU) in East China, which will provide more clinical evidence on the management of LVT.

## Materials and methods

### Research design and population

Between January 2010 and January 2020, 542,844 echo studies were screened from the echocardiography reporting system of the SAHZU. All patients with a reported LVT confirmed by 2 independent experts, regardless of the underlying disease, were included. One patient with right ventricular thrombus, 3 patients with atrial thrombus, and 9 patients lost to follow-up were excluded. All the included patients were followed up by phone call or at the outpatient clinic. This study has been approved by the Ethics Committee of SAHZU. The patients’ informed consent were exempted due to the nature of the study.

### Thrombus evaluation

Echocardiography analyses were performed for this study by an independent cardiologist in accordance with the published guidelines. Contrast transthoracic echocardiography (TTE) was performed to confirm the diagnosis of LVT if the initial TTE was inconclusive. To be distinguishable from the underlying myocardium, a clear thrombus–blood interface was required and the LVT had to be visible on at least 2 orthogonal views. The number, size (the largest two-dimensional area available on the index echocardiogram), location, and echogenicity of each thrombus were evaluated. All thrombus data were evaluated using the Philips EPIQ7 Ultrasound System (Philips Ultrasound, Inc.).

### Definition of end points

The primary endpoints were major adverse cardiovascular events (MACEs) and all-cause mortality. MACE was defined as all-cause mortality, ischemic stroke, acute MI, and acute peripheral artery emboli (4–6). The secondary end points were ischemic stroke, acute MI, and acute peripheral artery emboli. The primary safety end point were bleeding events defined as any clinically relevant moderate and severe bleeding events according to thrombolysis in myocardial infarction (TIMI) classification: TIMI I—intracranial hemorrhage or clinically visible hemorrhage (including imaging), with a decrease of hemoglobin concentration ≥ 5 g/dL and TIMI II—clinically visible hemorrhage with decreased hemoglobin concentration by 3–5 g/dL (7). Total LVT regression was defined by a complete disappearance of LVT on all echocardiography views at the last available follow-up (8).

### Data collection

We constructed a local database termed LVT database and used uniform standards by training to collect the data on socioeconomic status, previous and current medical histories, laboratory investigations, echocardiography, coronary angiography, and medication of the patients with LVT from the electronic medical record (EMR) system and ultrasonography system. Individual case report form was created to collect outpatient and phone call follow-up data. MACE and bleeding events (TIMI I and II) during the period of observation were recorded.

### Statistical analysis

All data were shown as mean ± standard deviation or median (interquartile range) for continuous variables and as the number (%) of patients for categorical variables. In order to identify independent correlates, the variables with a *p-*value < 0.05 in univariate analysis were entered into multivariate regression analysis using a forward likelihood-ratio method for MACE and all-cause mortality. The number of the other end points is small, resulting in a low incidence, which is not enough for the corresponding regression analysis, and no further analysis was performed. The 95% CI for HR was presented. A two-sided *p* < 0.05 was considered statistically significant. All statistical analyses were conducted using Statistical Package for the Social Sciences version 25.0 (SPSS Inc., Chicago, IL, United States) (9).

## Results

### Baseline clinical characteristics

A total of 237 patients diagnosed with LVT were definitively included, 26 patients further received left ventricular contrast TTE for the confirmation of LVT. The mean age was 59.9 ± 15.2 years, 84% were men. The baseline characteristics of patients are described in Table 1. In total, 168 patients (70.9%) had coronary heart disease, 28 patients (11.8%) had atrial fibrillation, 65 patients (27.4%) had heart failure (HF), and 23 patients (9.7%) had a history of stoke. Almost half of the population (48.9%) had a history of anterior wall MI, and 49 patients (20.7%) had a history of DCM.

**TABLE 1:** Baseline clinical characteristics and transthoracic echocardiography (TTE) findings.

Variable	All patients (*n* = 237)
Age(years)	59.89 ± 15.55
Male	199 (84%)
BMI (kg/m^2^)	23.76 ± 3.74
Smoking	110 (46.4%)
Hypertension	116 (48.9%)
Diabetes	42 (17.7%)
Hyperlipidemia	11 (4.6%)
Stroke	23 (9.7%)
Atrial fibrillation	28 (11.81%)
Coronary heart disease	168 (70.89%)
Previous PCI	133 (56.12%)
Previous CABG	9 (3.80%)
Anterior myocardial infarction	116 (48.9%)
Cardiomyopathy	55 (23.2%)
LA size (cm)	4.15 ± 0.70
LVIDd (cm)	5.70 ± 0.98
LVIDs (cm)	4.47 ± 1.20
LVEF (%)	40.05 ± 14.67
LVEF ≤ 50%	168 (70.89%)
Apex location	219 (92.4%)
Ventricular aneurysm	66 (27.85%)
Thrombus area (cm^2^)	2.76 (1.76-4.47)
Number of thrombus>1	27 (11.4%)

BMI, body mass index; PCI, percutaneous coronary intervention; CABG, caronary artery bypass grafting; LA, left atrium; LVIDd, left ventricular end-diastalic diameter; LVIDs, left ventricular end-systolic diameter; LVEF, left ventricular ejection fraction.

The detailed baseline echocardiographic parameters are shown in Table 1. In brief, the mean ejection fraction (EF) was 40.05 ± 14.67%, and the median value of the thrombus area was 2.76 (1.76–4.47) cm^2^. In total, 27 cases (11.4%) were complicated with more than two thrombi, 31 cases (13.08%) were with mobile thrombi, 157 cases (66.2%) were with moderate and high echogenicity thrombi; 46 (19.4%) thrombi were inside the aneurysm, 219 (92.4%) were located in heart apex (Table 1).

### Medication treatments

Most of the study population (82.3%) was treated with anticoagulation therapy, including vitamin K antagonists (VKA) (65.8%; *n* = 156), direct oral anticoagulants (DOACs) (12.7%; *n* = 30), and low molecular weight heparin (LMWH) (3.8%; *n* = 9). Anticoagulation + antiplatelet therapy was prescribed in 49.8% (*n* = 118) of patients. In total, 42 patients did not take any anticoagulants; of which, 38 cases (90.5%) took 1–2 kinds of antiplatelet drugs and 1 patient with subarachnoid hemorrhage, 1 patient with heart transplantation, and 2 patients refused to take anticoagulants (Table 2).

**TABLE 2:** Medication treatment following left ventricular thrombus diagnosis.

Variable	All patients (*n* = 237)
Antiplatelet therapy only	38 (16%)
Anticoagulation only	77 (32.5%)
Anticoagulation + antiplatelet therapy	118 (49.8%)
Aspirin + anticoagulant	28 (11.8%)
Clopidogrel/ticagrelor + anticoagulant	24 (10.1%)
Aspirin + clopidogrel/ticagrelor + anticoagulant	66 (27.8%)
Anticoagulation type	195 (82.3%)
Warfarin	156 (65.8%)
DOACs	30 (12.7%)
LMWH	9 (3.8%)
RASI	121(51.1%)
Aldosterone antagonist	115 (48.5%)
β-blocker	173 (73%)
Digoxin	46 (19.4%)

DOACs, direct oral anticoagulants; LMWH, low molecular weight heparin; RASI, renin angiotensin inhibitor.

### Follow-up results

#### Outcomes of the thrombus

Among 237 patients with LVT, 182 patients underwent follow-up TTE. Thrombus resolution was achieved in 152 cases (64.1%) with a median time of 57 days from the baseline echocardiography to the final echocardiography, and residual LVT was observed in 30 cases (12.7%). Among 55 patients who did not undergo follow-up TTE, death events were reported in 42 cases.

#### Outcomes of the events

Within a median follow-up period of 736 days, the rate of MACE occurred in 36.7% (*n* = 87): all-cause mortality 28.3%, ischemic stroke 8.4%, MI 3%, and peripheral artery emboli 1.7%. Of all-cause mortality, 61 cases (91%) were cardiovascular deaths and 6 patients (9%) died of non-cardiovascular origin, including pneumonia, cancers, bleeding, and multiple injuries. The median duration from diagnosis of LVT to death was 318 days. All emboli complications included 20 cases with stroke, 7 cases with MI, and 4 cases with peripheral artery emboli. Bleeding events of varying degrees occurred in 16.9% (*n* = 40) patients: TIMI I bleeding 4.2% (*n* = 10), TIMI II bleeding 3.4% (*n* = 8), and TIMI III bleeding 9.3% (*n* = 22) of patients, respectively. Bleeding events included 6 cases of cerebral hemorrhage, 14 cases of the gastrointestinal tract, 2 cases of hemoptysis, 2 cases of the urinary system, 1 case of the abdominal cavity, 2 cases of postoperative wound bleeding, and 17 cases of skin and mucous membranes (4 of them had bleeding in 2 sites). In addition, we also collected the following clinical outcomes during the period of observation: 5 cases had heart transplantation, 1 case underwent ventricular tumor resection, 1 case underwent ventricular aneurysm closure, 2 patients had a new left atrial thrombus, 4 cases had venous emboli, and 20 patients were observed to have recurrent LVT.

### Outcomes of statistical analysis

#### Logistic regression analysis for major adverse cardiovascular event

Univariate analysis showed that coronary heart disease (*p* = 0.017), atrial fibrillation (*p* = 0.020), left ventricular end-systolic diameter (LVIDs; *p* = 0.011), moderate and severe renal function injury (*p* = 0.009), left ventricular ejection fraction (LVEF) ≤ 50% (*p* = 0.006), and β-blocker use (*p* = 0.004) were significantly correlated to MACE. These variables were entered in multivariate logistic regression analysis using a forward likelihood-ratio method. Finally, atrial fibrillation (HR, 3.049; 95% CI 1.264–7.355; *p* = 0.013), moderate and severe renal function injury (HR, 2.097; 95% CI, 1.027–4.281; *p* = 0.042), and LVEF ≤ 50% (HR, 2.243; 95% CI 1.090–4.615; *p* = 0.028) were independent risk factors for MACE, whereas the use of β-blocker (HR, 0.397; 95% CI 0.210–0.753; *p* = 0.005) was a protective factor (Table 3).

**TABLE 3:** Logistic regression analysis for the association between major adverse cardiovascular events (MACEs) and clinical findings.

Variable	Univariate regression			Multivariate regression		
	*P*-value	HR	95% CI	*P*-value	HR	95% CI
Age	0.100	1.015	0.997–1.033	–	–	–
Male	0.985	0.993	0.484–2.039	–	–	–
BMI(kg/m^2^)	0.285	0.989	0.970–1.092	–	–	–
Smoking	0.622	1.143	0.672–1.943	–	–	–
Alcohol	0.875	0.947	0.479–1.870	–	–	–
Hypertension	0.989	1.004	0.592–1.702	–	–	–
Diabetes	0.617	0.836	0.413–1.690	–	–	–
Hyperlipidemia	0.086	0.163	0.200–1.294	–	–	–
Stoke	0.480	1.369	0.573–3.268	–	–	–
Cardiomyopathy	0.675	1.111	0.679–1.819	–	–	–
Coronary heart disease	**0.017**	0.521	0.305–0.890	–	–	–
Atrial fibrillation	**0.020**	2.592	1.163–5.775	**0.013**	3.049	1.264–7.355
Previous CABG	0.074	3.630	0.884–14.899	–	–	–
Previous PCI	0.065	0.605	0.355–1.031	–	–	–
Moderate and severe renal dysfunction	**0.009**	2.462	1.258–4.818	**0.042**	2.097	1.027–4.281
LA size	0.573	1.114	0.765–1.621	–	–	–
LVIDd	0.200	1.194	0.910–1.567	–	–	–
LVIDs	**0.011**	1.340	1.071–1.677	–	–	–
LVEF ≤ 50%	**0.006**	2.425	1.282–4.586	**0.028**	2.243	1.090–4.615
Ventricular aneurysm	0.063	0.554	0.298–1.032	–	–	–
Thrombus Area(cm^2^)	0.100	1.054	0.990–1.122	–	–	–
Number of thrombus >1	0.419	0.698	0.292–1.670	–	–	–
Antiplatelet therapy only	0.940	0.979	0.561–1.707	–	–	–
Anticoagulation only	0.617	1.197	0.592–2.142	–	–	–
Anticoagulation + antiplatelet therapy	0.854	1.051	0.620–1.782	–	–	–
RASI	0.084	0.626	0.368–1.066	–	–	–
Aldosterone antagonist	0.806	1.936	0.550–1.591	–	–	–
β-blocker	**0.004**	0.426	0.237–0.766	**0.005**	0.397	0.210–0.753
Digoxin	0.955	1.019	0.523–1.988	–	–	–

BMI, body mass index; CABG, coronary artery bypass grafting; PCI, percutaneous coronary intervention; LA, left atrium; LVIDd, left ventricular end-diastalic diameter; LVIDs, left ventricular end-systolic diameter; LVEF, left ventricular ejection fraction; RASI, renin angiotensin inhibitor. Bold values are statistical differences more eye-catching.

#### Cox regression analysis for all-cause mortality

**FIGURE 1:**
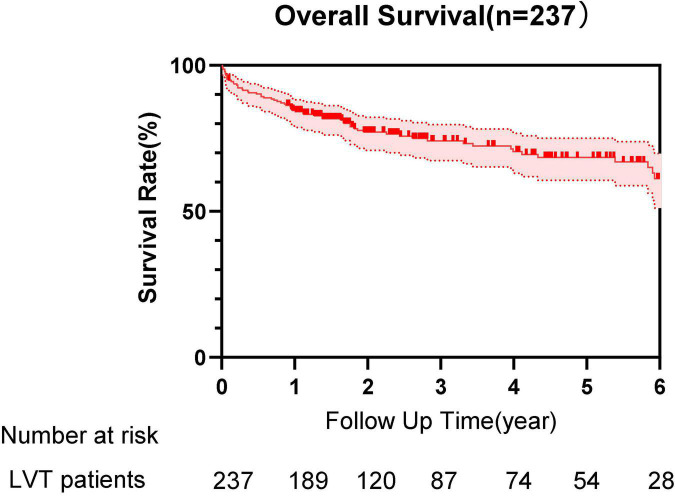
Survival curve of patients with left ventricular thrombus (LVT) during the follow-up time.

Univariate analysis showed that age (*p* = 0.018), male (*p* = 0.035), previous caronary artery bypass grafting (CABG; *p* = 0.006), previous percutaneous coronary intervention (PCI; *p* = 0.007), LVIDs (*p* = 0.001), LVEF ≤ 50% (*p* < 0.001), thrombus area (*p* = 0.029), and β-blocker use (*p* = 0.001) were significantly correlated to all-cause mortality. These variables were entered in multivariate cox regression analysis using a forward likelihood-ratio method. Finally, age (HR, 1.021; 95% CI 1.002–1.040; *p* = 0.031), previous CABG (HR, 4.634; 95% CI, 2.042–10.517; *p* < 0.001), LVEF ≤ 50% (HR, 3.714; 95% CI 1.664–8.290; *p* = 0.001), and thrombus area (HR, 1.071; 95%CI, 1.019–1.126; *p* = 0.007) were independent risk factors for all-cause mortality, whereas the use of β-blocker (HR, 0.410; 95% CI 0.237–0.708; *p* = 0.001) was a protective factor (Table 4 and Figure 1).

**TABLE 4:** Cox regression analysis for the association between all-cause mortality and clinical findings.

Variable	Univariate regression			Multivariate regression		
	*P*-value	HR	95% CI	*P*-value	HR	95% CI
Age	**0.018**	1.02	1.003–1.037	**0.031**	1.021	1.002–1.040
Male	**0.035**	0.755	0.418–1.361	–	–	–
BMI	0.975	0.898	0.970–1.060	–	–	–
Smoking	0.957	1.013	0.623–1.649	–	–	–
Hypertension	0.861	0.958	0.593–1.548	–	–	–
Diabetes	0.981	1.008	0.54–1.882	–	–	–
Hyperlipidemia	0.916	0.272	0.38–1.958	–	–	–
Stoke	0.689	0.842	0.364–1.950	–	–	–
Alcohol	0.868	0.947	0.495–1.808	–	–	–
Cardiomyopathy	0.210	1.308	0.859–1.991	–	–	–
Coronary heart disease	0.055	2.516	0.981–6.452	–	–	–
Atrial fibrillation	0.105	1.676	0.898–3.131	–	–	–
Previous CABG	**0.006**	2.981	1.360–6.536	** < 0.001**	4.634	2.042–10.517
Previous PCI	**0.007**	0.528	0.311–0.831	–	–	–
Moderate and severe renal function injury	0.984	1.006	0.579–1.748	–	–	–
LA size	0.347	1.175	0.839–1.646	–	–	–
LVIDd	0.060	1.261	0.99–1.606	–	–	–
LVIDs	**0.001**	1.354	1.125–1.629	–	–	–
LVEF ≤ 50%	**<0.001**	4.753	2.169–10.418	**0.001**	3.714	1.664–8.290
Ventricular aneurysm	0.061	0.559	0.305–1.027	–	–	–
Thrombus area (cm^2^)	**0.029**	0.52	1.005–1.101	**0.007**	1.071	1.019–1.126
Number of thrombus >1	0.263	0.619	0.267–1.435	–	–	–
Antiplatelet therapy only	0.495	0.797	0.416–1.529	–	–	–
Anticoagulation only	0.865	1.045	0.627–1.744	–	–	–
Anticoagulation + antiplatelet therapy	0.702	1.098	0.680–1.774	–	–	–
RASI	0.953	1.015	0.621–1.660	–	–	–
Aldosterone antagonist	0.656	1.116	0.688–1.809	–	–	–
β-blocker	**0.001**	0.464	0.285–0.754	**0.001**	0.410	0.237–0.708
Digoxin	0.595	1.174	0.65–2.119	–	–	–

BMI, body mass index; CABG, coronary artery bypass grafting; PCI, percutaneous coronary intervention; LA, left atrium; LVIDd, left ventricular end-diastalic diameter; LVIDs, left ventricular end-systolic diameter; LVEF, left ventricular ejection fraction; RASI, renin angiotensin inhibitor. Bold values are statistical differences more eye-catching.

## Discussion

### Clinical characteristics

Despite adequate interventional and medical therapy, the incidence of LVT is still high in both ischemic and non-ischemic cardiomyopathies currently. LVT remains to be an important cause for cerebral and peripheral arterial embolism and subsequent mortality. Patients with LVT are with poor clinical prognosis and high risks of MACE. This study provides new insights into the clinical characteristics and prognosis from a relatively large cohort of patients with LVT in the Chinese population.

The average age of 237 patients enrolled in this study is 59.9 ± 15.2 years, which is similar to the age reported abroad. The incidence of women is significantly lower compared with men, the proportion of women in our hospital (16%) is similar to the proportion of women reported abroad (15–30.4%) (10, 11). Coronary heart disease is still the main disease complicated by LVT in this study, but when compared with the study by Lee et al. (3), the rate of coronary heart disease-related LVT was decreased (70.9% vs. 80.6%). This may be due to the progress of PCI technology and the normalization of various medication treatments, such as anti-platelet aggregation and anti-arteriosclerosis for coronary heart disease.

In this study, DCM, valvular heart disease, and other concurrent LVT diseases increased by 9.7% when compared with the study by Lee et al. (3), which may be related to the advance in thrombus detection technology, the prolongation of the population’s average life-span, and the better healthcare nowadays.

Transthoracic echocardiography remains the main method for detecting LVT due to its convenience, non-invasiveness, strong reproducibility, and high specificity. The advances in ultrasound technology and the use of contrast agents potentially help clinicians to identify LVT (12). In this study, the thrombus was mostly located in the apex of the left ventricle where blood flow was the slowest or most stagnant due to abnormal ventricular wall movement, and thrombus was found in 52% of ventricular aneurysms. Thrombus echogenicity was dominated by medium-to-high echogenicity (66.2%), indicating the high degree of thrombus calcification.

### Anticoagulation therapy

At present, due to the lack of clinical evidence and considering the bleeding risk of combined application of antiplatelet and anticoagulant medication, there are certain controversies about the treatment strategy for LVT caused by ischemic heart disease. STEMI guidelines recommend additional anticoagulation on the basis of antiplatelet treatment in patients developing LVT, with VKA as the standard anticoagulant agent. The 2013 ACC/AHA guideline for STEMI management suggested adding VKA to dual antiplatelet therapy (DAPT) in patients with LVT for at least 3 months (13). Similarly, the 2014 ASA guideline for primary prevention of stroke gives an IIa recommendation for using VKA adjunctive to DAPT in STEMI patients with LVT (14). The 2017 ESC guidelines for STEMI recommend treatment of LVT with oral anticoagulation for up to 6 months guided by repeated imaging, but no agent preference is given (15). In 2018, CCS issued guidelines for antiplatelet therapy: for the treatment of patients with LVT after PCI, it is recommended to use aspirin, clopidogrel, and oral anticoagulants for initial treatment, but stop aspirin within 6 months (16). A total of 168 patients with ischemic heart disease complicated with LVT were enrolled, 37 of whom did not take anticoagulants, accounting for 22%. During the first half of the decade, 36.4% of patients did not take anticoagulants. While from February 2015 to January 2020, 15% of patients did not take anticoagulants, so clinicians are more active in the anticoagulation treatment of LVT caused by ischemic heart disease.

The anticoagulant treatment plan for LVT caused by non-ischemic heart disease has been relatively clear. Patients with DCM with LVEF < 30% or a history of embolism or echocardiography found mural thrombosis is recommended to add treatment with anticoagulants. In this study, a total of 38 patients with DCM with LVEF < 30%, 2 of whom did not take anticoagulants due to waiting for heart transplantation. Therefore, patients with DCM in this study nearly meet the guideline-directed anticoagulation treatment plan.

However, the total rate of anticoagulant treatment was 82.3% in this study, which was a little low compared with 98.7% in a similar study by Lattuca et al. (8) in the United States (8). Therefore, the treatment of LVT in China is still more conservative. In a study of 244 patients with MI complicated with LVT, the median follow-up time was 807 days, and the thrombus disappearance rate was 63.96% (12). In another study, 156 patients with all diseases complicated with LVT were followed up for a median of 632 days, and the thrombus disappeared by 66.7%, compared with 64.1% in our study (8). These studies highlight that the current antithrombotic regimen needs to be improved because nearly one-third of patients did not achieve total LVT regression and remained exposed to a high risk of clinical complications even when combined with antiplatelet agents.

Fortunately, since 2020, there is an increasing number of studies done to explore more reasonable anticoagulation schemes in the treatment of LVT. Lots of articles discussed the comparison of the effects of DOACs and VKA, which showed no significant difference in the incidence of new thromboembolic events, bleeding, the rate of resolution of thrombus, and even the all-cause mortality. DOACs and VKA have similar efficacy and safety in treating LVT, prompting the inference that DOACs are the possible alternatives to VKA in LVT therapy. Most recently, the breakthrough of 2 novel randomized controlled trials have shown DOACs to be a promising treatment for LV thrombus. They also appealed that the optimal timing and type of anticoagulation for LV thrombus, and the role of screening for high-risk patients, should be tested in more prospective, randomized trials (17–25). We analyzed the relationship between baseline medication and mortality within 1 month and found aspirin/clopidogrel/ticagrelor + anticoagulant (HR, 0.066; 95% CI 0.011–0.403; *p* = 0.003) and aspirin + clopidogrel/ticagrelor + anticoagulant (HR, 0.059; 95% CI 0.004–0.804; *p* = 0.034) had protective effect on mortality. It indicated that the baseline medication has an impact on mortality within 1 month.

### Clinical outcomes

The main result of this study showed a high rate of MACE in patients with LVT, as 28.3% of patients died and 13% of patients had embolic complications during follow-up. A study by Lattuca et al. (8) from Europe reported that the mortality and embolic complications occurred in 18.9% (*n* = 30) and 22.2% (*n* = 35) of 156 patients, respectively (8). A study from Singapore showed that the all-cause mortality rate was 21.7% (*n* = 53) of 244 patients with post-AMI LVT (12). Meanwhile, a study from Xinqiao Hospital in China showed that the mortality and the embolic complications rate of 92 patients were 30.4 and 10.9%, respectively, within a median follow-up period of 702 days (10). Another study from Shanghai East Hospital showed that after following up for 1 year, the frequency of mortality and embolic complications was 12 and 28%, respectively, for 25 patients with post-MI LVT (26). Based on these studies, the MACE of patients with LVT is especially high nationally and internationally. The mortality of our patients is higher than other studies. It may be due to the availability of NOACs, alertness, inertia of clinician, and longer follow-up time. Cox regression analysis in this study showed that those who underwent CABG surgery before the formation of thrombus had a 4.634 times higher risk of death than those who did not. Meanwhile, it also showed the risk of death for patients with LVEF ≤ 50% was 3.714 times higher than the patients with LVEF > 50%, and the risk of death increased 1.071 times for every 1 cm^2^ increase in thrombus area. These results had also been confirmed by other studies. In this way, we supposed that relatively aggressive treatment could be considered for patients with severe coronary heart disease or lower LVEF or bigger LVT area in order to improve the prognosis of these patients (9–12, 26, 27).

In addition, according to the 2017 AHA/ACC/HRS Guideline for Management of Patients with Ventricular Arrhythmias and the Prevention of Sudden Cardiac Death, β-blockers are generally safe agents that effectively suppress ventricular ectopic beats and arrhythmia and prevent sudden cardiac death in a wide array of cardiac diseases. According to the guidelines, β-blockers are indicated in all patients, except those with AV block, bradycardia, or asthma and are recommended in all patients with HF, regardless of baseline rhythm, and β-blockers are also used for the control of ventricular rates to avoid rapid irregular ventricular activation (28, 29). In this study, the risk of MACE for patients taking the medication of β-blocker was reduced to nearly one-third compared to that who did not take it, which was consistent with the above guidelines.

## Study limitations

This study is a retrospective study from a single center. The research was conducted based on a retrospective observation and analysis of data collected in a tertiary hospital located in East China. We could not exclude the influence of geographical, economic, and cultural differences. Most patients only underwent TTE examinations. Due to its limited sensitivity in detecting LVT, the detection rate of LVT in this study may be underestimated. Due to the nature of the retrospective study, the study was not conducted regularly with continuous TTE to determine more accurate LVT resolution time and the possibility of LVT recurrence after stopping treatment; there may be unmeasured variables in the study, which may be important predictors of LVT. In addition, the information, especially for the medication data, that is provided by the phone call follow-up recipients by memories maybe not completely accurate, which leaves room for uncertainty in our research results (30). Despite these limitations, this study provides valuable data for the clinical characteristics, treatment, and prognosis of LVT in China.

## Conclusion

Most studies discuss the risk factors for LVT formation, whereas our study focuses on the risk factors of MACE and all-cause mortality after LVT formation. This study showed that atrial fibrillation, moderate and severe renal function injury, and LVEF ≤ 50% were independent risk factors for MACE; age, previous CABG, LVEF ≤ 50%, and large thrombus area were independent risk factors for all-cause mortality. It was found that the use of β-blockers could improve prognosis for the first time. LVT is an uncommon complication of ischemic and non-ischemic cardiomyopathy, which is associated with a high risk of adverse events and mortality. It is recommended that doctors could be more active in applying patients with LVT with anticoagulants. More randomized controlled studies with a large sample size should be performed to assess the efficacy and safety of target-specific treatment for patients with LVT.

## Data availability statement

The original contributions presented in this study are included in the article/supplementary material, further inquiries can be directed to the corresponding authors.

## Ethics statement

This study has been approved by the Ethics Committee of SAHZU. Due to the nature of the study, written informed consent was not obtained from the individual(s) for the publication of any potentially identifiable images or data included in this article.

## Author contributions

CL involved in data collection, collation, follow-up, and statistical analysis and wrote the original draft. WL involved in data collection, collation, and follow-up and helped in the drafting of the manuscript. NQ involved in statistical analysis, contributed to the interpretation of data, and revised the manuscript. LS involved in data collection and follow-up. CJ involved in revising manuscript. DZ and YY involved in statistical analysis. XP involved in the conception, research design, and revision of manuscript and helped in the acquisition of data. QZ involved in the study design and final revision of manuscript. All authors contributed to the article and approved the submitted version.
